# The complete mitochondrial genome of *Comaster schlegelii* (Crinoidea, Comatulida)

**DOI:** 10.1080/23802359.2021.2008819

**Published:** 2022-02-03

**Authors:** Yuyao Sun, Xiaomei Liao, Yue Dong, Sudong Xia, Qinzeng Xu

**Affiliations:** a Tianjin Key Lab of Aqua-Ecology and Aquaculture, Fisheries College, Tianjin Agricultural University, Tianjin, China; b MNR Key Laboratory of Marine Eco-Environmental Science and Technology, First Institute of Oceanography, Ministry of Natural Resources, Qingdao, China

**Keywords:** Crinoidea, Comatulidae, *Comaster schlegelii*, Mitochondrial genome

## Abstract

*Comaster schlegelii*, belonging to the family Comatulidae, is a variable feather star distributed in the Pacific Ocean. The complete mitochondrial genome of this species was 15,887 bp in length, consisting of 13 protein-coding genes, 22 transport RNA genes, two ribosomal RNA genes and one control region. The whole mitochondrial genome of *C. schlegelii* had a high AT content of 72.73%. The phylogenetic relationship was reconstructed with 16 relevant echinoderms, which revealed *C. schlegelii* was closely clustered with *Anneissia pinguis* in the family Comatulidae.

*Comaster schlegelii* (Carpenter, 1881), variable bushy feather star with multicolor, is a crinoid in the order Comatulida, family Comatulidae. This species is common in the shallow-water regions, especially in the coral reefs of the western Pacific Ocean (Summers et al. 2017). In accordance with other feather stars, *C. schlegelii* is a suspension feeder and spreads out its arms and pinnules to feed. The phylogenetic relationships within Crinoidea are controversial and lack sufficient molecular evidence to validate. Till now, as for the progress of mitochondrial genome research, only nine species from four families were published, including the family Antedonidae (Nam et al. 2020), Sclerocrinidae (Scouras and Smith 2006), Comatulidae (Scouras and Smith 2006) and Mariametridae (Ma et al. 2019). For the family Comatulidae, only two mitochondrial genomes from the genus *Anneissia* (Kim and Shin 2021) and *Phanogenia* (Scouras and Smith 2006) were reported. Therefore, we sequenced the mitochondrial genome of *C. schlegelii* and enrich the comprehension of the genetic phylogenetic relationship of the Comatulidae family compared with other echinoderms.

The *C. schlegelii* sample was collected from Wuzhizhou Island in Sanya, China (18.31°N, 109.76°E, ≈ 12 m). The tissue sample was preserved in MNR Key Laboratory of Marine Eco-Environmental Science and Technology, First Institute of Oceanography, Ministry of Natural Resources (NO. FIO-ECH-HBH01). The genomic DNA of *C. schlegelii* was extracted using QIAamp Fast DNA Tissue Kit (QIAGEN, Germany). After quality control, the qualified DNA was sheared, processed to prepare 350 bp DNA library and pair-end sequenced on the Illumina Novaseq 5000 platform (PE150, 10×) in the Biomarker Corporation (Beijing, China).

The raw data were trimmed by Trimmomatic v0.38 (Bolger et al. 2014), and the filtered data were assembled using SPAdes v3.6.1 (Bankevich et al. 2012). Nucleotide Blast was performed with other species from Crinoidea in GenBank as the references to query the fragments of mitochondrial genome. Geseq (Tillich et al. 2017) and MITOs (Hahn et al. 2013) were used for preliminary annotation, and tRNA was annotated by tRNA scan-SE (Lowe and Chan 2016). The alignment and correction of amino acids were performed according to MEGA v7.0 software (Kumar et al. 2016) and the position of coding genes was corrected by Unipro UGENE (Okonechnikov et al. 2012). The complete mitochondrial genome was submitted to GenBank with accession number MW526391.

The complete mitochondrial genome for *C. schlegelii* was 15,887 bp in length, including 13 protein-coding genes (PCGs), 22 tRNA genes, 2 rRNA genes, and one control region. The overall base composition was 26.73% A, 11.38% C, 15.89% G, and 46.00% T. The gene order of *C. schlegelii* was consistent with other species from the Comatulidae, indicating that the gene order was conserved in this family.

A Maximum Likelihood tree was constructed by MEGA v7.0 software (Kumar et al. 2016) using the dataset containing 13 PCGs and 2 rRNA. The nine published mitochondrial genomes from Crinoidea were chosen and the seven outgroups were from other classes. The phylogenetic tree depicted that *C. schlegelii* formed a branch with *A. pinguis* (Figure 1), as the representative of the family Comatulidae. They clustered together with other feather stars to form the class Crinoidea. This mitochondrial genome analysis provides the foundation for phylogeny studies in the genus *Comaster*. Further studies call for more mitochondrial genomes resources to explore the relationship within Crinoidea.

**Figure 1.**
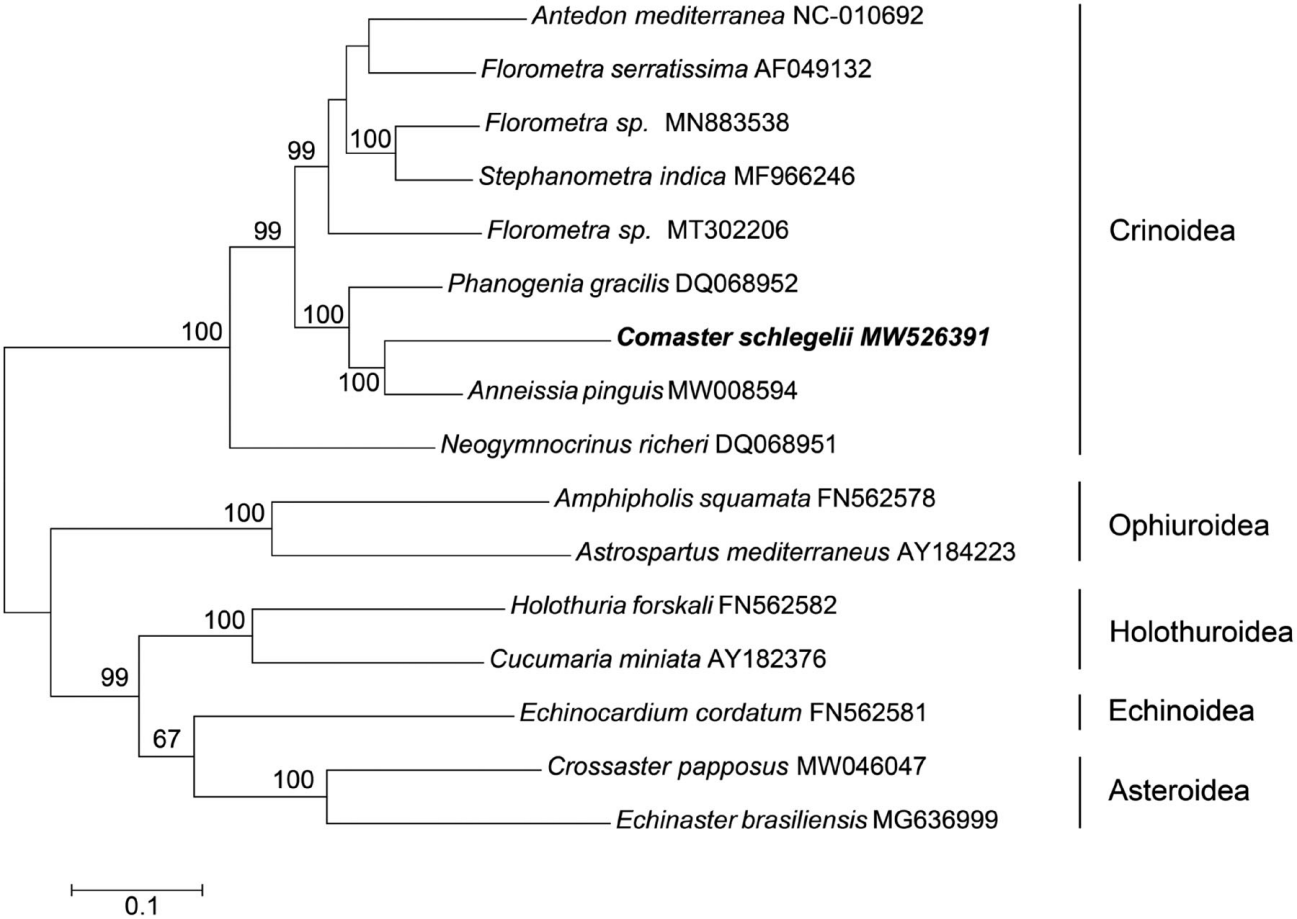
Maximum-likelihood tree for the feather star *Comaster schlegelii* and GenBank representatives of the phylum Echinodermata. The tree was constructed using 13 protein-coding genes and 2 rRNA. The tree was based on the Kimura 2-parameter model of nucleotide substitution. The numbers at the nodes are bootstrap percent probability values based on 1000 replications. The mitochondrial genome sequence obtained at the present study was indicated in bold type.

## Data Availability

The data that support the findings of this study are openly available in the National Center for Biotechnology Information (NCBI) at https://www.ncbi.nlm.nih.gov. The accession number of the complete mitochondrial genome is MW526391. The associated BioProject, SRA, and Bio-Sample numbers are PRJNA692719, SUB8902528 and SAMN17367682, respectively.
